# Genetic Variants and Somatic Alterations Associated with MITF-E318K Germline Mutation in Melanoma Patients

**DOI:** 10.3390/genes12091440

**Published:** 2021-09-18

**Authors:** Elisabetta Vergani, Simona Frigerio, Matteo Dugo, Andrea Devecchi, Erika Feltrin, Loris De Cecco, Viviana Vallacchi, Mara Cossa, Lorenza Di Guardo, Siranoush Manoukian, Bernard Peissel, Andrea Ferrari, Gianfrancesco Gallino, Andrea Maurichi, Licia Rivoltini, Marialuisa Sensi, Monica Rodolfo

**Affiliations:** 1 Unit of Immunotherapy, Department of Research, Fondazione IRCCS Istituto Nazionale dei Tumori, Via Venezian 1, 20133 Milan, Italy; elisabetta.vergani@istitutotumori.mi.it (E.V.); simona.frigerio@istitutotumori.mi.it (S.F.); viviana.vallacchi@istitutotumori.mi.it (V.V.); licia.rivoltini@istitutotumori.mi.it (L.R.); 2 Platform of Integrated Biology, Department of Applied Research and Technology Development, Fondazione IRCCS Istituto Nazionale dei Tumori, Via Amadeo 42, 20133 Milan, Italy; Dugo.Matteo@hsr.it (M.D.); andrea.devecchi@istitutotumori.mi.it (A.D.); loris.dececco@istitutotumori.mi.it (L.D.C.); marialuisa.sensi@istitutotumori.mi.it (M.S.); 3 CRIBI Biotechnology Center, Via Bassi 58/B, 35131 Padua, Italy; eri.feltrin@gmail.com; 4 Department of Pathology, Fondazione IRCCS Istituto Nazionale dei Tumori, Via Venezian 1, 20133 Milan, Italy; mara.cossa@istitutotumori.mi.it; 5 Department of Medical Oncology, Fondazione IRCCS Istituto Nazionale dei Tumori, Via Venezian 1, 20133 Milan, Italy; lorenza.diguardo@istitutotumori.mi.it; 6 Unit of Medical Genetics, Fondazione IRCCS Istituto Nazionale dei Tumori, Via Venezian 1, 20133 Milan, Italy; Siranoush.Manoukian@istitutotumori.mi.it (S.M.); bernard.peissel@istitutotumori.mi.it (B.P.); 7 Pediatric Oncology Unit, Fondazione IRCCS Istituto Nazionale dei Tumori, Via Venezian 1, 20133 Milan, Italy; andrea.ferrari@istitutotumori.mi.it; 8 Melanoma and Sarcoma Surgery Unit, Fondazione IRCCS Istituto Nazionale dei Tumori, Via Venezian 1, 20133 Milan, Italy; gianfranco.gallino@istitutotumori.mi.it (G.G.); andrea.maurichi@istitutotumori.mi.it (A.M.)

**Keywords:** cutaneous melanoma, MITF gene, MITF-E318K variant, germline mutations, somatic mutations, copy number alterations, MTAP gene, SKCM-TCGA Pan Cancer Atlas

## Abstract

The MITF-E318K variant has been implicated in genetic predisposition to cutaneous melanoma. We addressed the occurrence of MITF-E318K and its association with germline status of CDKN2A and MC1R genes in a hospital-based series of 248 melanoma patients including cohorts of multiple, familial, pediatric, sporadic and melanoma associated with other tumors. Seven MITF-E318K carriers were identified, spanning every group except the pediatric patients. Three carriers showed mutated CDKN2A, five displayed MC1R variants, while the sporadic carrier revealed no variants. Germline/tumor whole exome sequencing for this carrier revealed germline variants of unknown significance in ATM and FANCI genes and, in four BRAF-V600E metastases, somatic loss of the MITF wild-type allele, amplification of MITF-E318K and deletion of a 9p21.3 chromosomal region including CDKN2A and MTAP. *In silico* analysis of tumors from MITF-E318K melanoma carriers in the TCGA Pan-Cancer-Atlas dataset confirmed the association with BRAF mutation and 9p21.3 deletion revealing a common genetic pattern. MTAP was the gene deleted at homozygous level in the highest number of patients. These results support the utility of both germline and tumor genome analysis to define tumor groups providing enhanced information for clinical strategies and highlight the importance of melanoma prevention programs for MITF-E318K patients.

## 1. Introduction

The microphthalmia-associated transcription factor (MITF) is a master regulator of melanocytes. Besides its key role in signaling via Melanocortin-1 Receptor (MC1R) upon binding by α-Melanocyte Stimulating Hormone to induce melanin production, MITF governs the maintenance of melanocyte stem cells, cell cycle progression, differentiation, apoptosis, migration, DNA damage repair, chromosome stability and melanoma pathogenesis [1,2]. The critical role of MITF and the complexity of its regulatory effects become evident in melanoma, where high MITF expression is associated with a differentiated and proliferative phenotype, whereas low MITF expression with dedifferentiation and invasion, but also with cell senescence [3,4,5]. MITF gene acts as an oncogene and shows gene amplification and gain of function mutations in metastatic melanomas [6]. MITF plays a complex role in the response to targeted therapies given that both increased as well as decreased MITF expression can mediate resistance to BRAF and MEK inhibitor therapy [7,8]. Recent evidence also indicates that MITF can impact the anti-melanoma immune responses [9]. As a reaction to stimuli from the microenvironment, melanoma cells can dynamically switch between invasive (MITF-low) and proliferative (MITF-high) phenotypes to promote cell plasticity, tumor heterogeneity and resistance to therapy [3,10].

The rare functional variant E318K (rs149617956) of MITF gene prevents SUMOylation at the lysine residue K316 and confers an increased risk for cutaneous melanoma (CM) [11,12]. SUMO is a small ubiquitin-like peptide that is coupled to target proteins to modify their functions, subcellular localization, or interaction with partner proteins [13]. The reduction of MITF SUMOylation regulates the expression of a large repertoire of genes involved in differentiation, pigmentation, cell growth, proliferation and movement, and can impair BRAF-V600E-induced senescence, although the complexity of the post-translational regulation of MITF remains largely undefined [1,14,15].

The MITF-E318K variant is mainly found in individuals of European descent and has been included in the list of moderate risk alleles predisposing to CM in both sporadic and familial melanoma [16]. It has also been shown to have a significant effect on nevus count and pigmentation [17,18,19,20]. Compared to non-carriers, MITF-E318K carriers have a higher risk of developing CM in association with other tumors, including pancreatic and renal cancer [11,21], while there is minimal evidence of MITF-E318K contribution to non-melanoma cancer risk [22]. MITF-E318K carriers are more prone to developing nodular or fast-growing melanoma [17,20,21,23,24,25,26], potentially reflecting the pathogenic effect of the mutant. Former studies have reported the occurrence of MITF-E318K carriers in the Italian population of CM patients, with a frequency of about 2%. MITF-E318K was observed not only in familial and multiple CM cases, in association or not to concurrent pathogenic variants of the Cyclin Dependent Kinase Inhibitor 2A gene (CDKN2A), but also in sporadic cases, and in some CM cases showing association to other cancer types [21,24,27,28,29]. 

Here we report the occurrence of the MITF-E318K germline variant in 7/248 CM patients of a hospital-based cohort, which was screened for the germline status of CDKN2A and MC1R predisposition genes, and included multiple (*n* = 78), familiar (*n* = 74), sporadic (*n* = 59), patients diagnosed for melanoma and other tumors (*n* = 15), and pediatric cases (*n* = 22). One sporadic melanoma MITF-E318K carrier resulted wild-type for CDKN2A and MC1R genes and displayed only germline variants of unknown significance in ATM and Fanconi anemia complementation group I (FANCI) genes. In addition, to unravel common somatic events in MITF-E318K sporadic melanomas, we performed genomic analysis using whole exome sequencing data of tumors from this patient and from the carriers included in the publicly available Skin Cutaneous Melanoma (SKCM) Pan Cancer Atlas collection dataset from The Cancer Genome Atlas (TCGA).

## 2. Materials and Methods

### 2.1. Patients

A consecutive series of 248 CM patients recruited between 1998 and 2015 for diagnostic and research purposes at the Fondazione IRCCS Istituto Nazionale dei Tumori were included. Patients underwent genetic testing for MITF, MC1R, CDKN2A germline variants in our laboratory. The studied case set encompasses CM patients with multiple primary melanoma (MM), with melanoma and other tumors (MN), with a melanoma family history (FM), children and adolescents (PM) and sporadic melanoma patients (SM), as detailed in Table 1. All the studies were approved by the Institutional Review Board and the Independent Ethics Committee and written informed consent was obtained from participants.

### 2.2. Genotyping of MITF, MC1R and CDKN2A 

MITF, MC1R and CDKN2A were analyzed on genomic DNA extracted from peripheral blood with the QiAamp DNA Blood Mini Kit (Qiagen, Redwood City, CA, USA). The screening for the MITF-E318K variant was carried out by polymerase chain reaction restriction fragment length polymorphism (PCR-RLFP) technique, taking advantage of a XmnI restriction site occurring in exon 9 wild type sequence (Figure S1). The identified carriers were confirmed by sequence analysis. Genotyping of CDKN2A and MC1R genes was carried out by sequence analysis according to published protocols [30,33]. PCR products were purified by ExoSAP-IT (USB Corporation, Cleveland, OH, USA) and sequenced by Eurofins Sequencing Service (Eurofins Genomics Germany GmbH, Ebersberg bei München, Germany). Sequences were analyzed by ChromasPro software (Technelysium Pty Ltd., South Brisbane, QLD, Australia). MC1R variants were classified as high-risk (R) and low-risk (r) variants as previously described [31,32].

### 2.3. Whole Exome Sequencing (WES)

Genomic DNA was extracted from peripheral blood and fresh frozen tumor tissues with DNeasy Blood and Tissue Kits with QIAcube automated station (Qiagen) following manufacturer’s instructions. DNA was quantified using the Qubit dsDNA high-sensitivity Assay kit (ThermoFisher Scientific, Waltham, MA, USA) and its quality was assessed by TapeStation 4200 (Agilent Technologies, Santa Clara, CA, USA). The Ion AmpliSeq Exome RDY kit (Thermo Fisher Scientific) was used for library prep according to manufacturer’s protocol. Briefly, 100 ng genomic DNA was used as starting material; pooled amplicons were end-repaired, and Ion Torrent adapters and amplicons were ligated with DNA ligase; sample barcoding was performed using the Ion DNA Barcoding kit. After purification using AMPure XP magnetic beads (Beckman Coulter, Brea, CA, USA), the concentration of the libraries was determined using the Ion Library Quantitation (Thermo Fisher Scientific). Template preparation and sequencing were performed at CRIBI Biotechnology Center of the University of Padua (Padua, Italy). Libraries were pooled, amplified by emulsion PCR, and enriched, with the One Touch and the ES machines (Thermo Fisher Scientific). For the sequencing, libraries were loaded into an PI Chip kit v3 chips and sequenced on the Ion Torrent Proton (Thermo Fisher Scientific) according to the manufacturer’s instructions. 

### 2.4. Bioinformatics Processing of WES Data

Both germline and tumor reads were aligned to the reference human genome sequence hg19 and coverage analysis was conducted using Ion Torrent Software (ITS). The AmpliSeq Exome files (BAM) were uploaded from Ion ITS to local Ion Reporter. Germline mutations were identified using AmpliSeq Exome single sample Germline workflow (version 5.6). A preliminary variant analysis was performed by using QueryOR [34], a platform for variant prioritization developed at CRIBI Biotechnology Center of the University of Padova (Padova, Italy). We focused the germline analysis on a list of 113 genes (Table S1) including well-established melanoma predisposing genes as well as genes reported in landmark studies of cancer predisposition [35,36,37,38,39,40]. Classification by COSMIC Cancer Gene Census is also provided [41]. The following selection criteria were applied: allele frequency in the ExAC database < 0.015, considered pathogenic by at least two prediction software (among SIFT, CADD, Fathmm, LRT, LR, Mutation Assessor, Mutation Taster, Radial SVM and Polyphen2), not referred to as benign or likely benign in ClinVar [42]. Somatic SNV and small indels were identified using AmpliSeq Exome tumor-normal workflow (version 5.6) with these modified parameters: “hotspot_min_allele_freq”: 0.03, “indel_min_allele_freq”: 0.02, “mnp_min_allele_freq”: 0.02 and “snp_min_allele_freq”: 0.02. To reduce possible false positive alterations, mutations were further filtered by removing variants matching at least one of the following filters: Phred score for quality mapping < 30, strand bias *p*-value < 0.01, normal coverage < 10, normal coverage ≥ 5 AND variant allelic frequency in normal > 0.1, tumor coverage < 200 AND variant allelic frequency in tumor < 0.1, number of reads supporting variant in tumor < 7. Only variants assumed to have a disruptive impact in the protein, probably causing protein truncation, loss of function or triggering nonsense-mediated decay by SIFT and Mutation Assessor, two algorithms that predict the effect of amino acid substitution on protein function were further considered. Called variants were summarized, analyzed, annotated, and visualized using the maftools Bioconductor package [43]. Somatic Copy Number Alterations (CNAs) from WES data were identified using EXCAVATOR2 [44]. To determine the frequency of somatic alterations in SKCM samples, the TCGA Pan Cancer Atlas dataset at the cBioPortal for Cancer Genomics repository (https://www.cbioportal.org/, accessed on 1 June 2021) [45,46], consisting of 488 samples of which 440 profiled for mutations and 367 for CNAs, was employed. 

## 3. Results

### 3.1. Genetic Testing for MITF-E318K, CDKN2A and MC1R in Melanoma Cohorts 

We genotyped for germline MITF-E318K mutation a series of 248 CM patients characterized for CDKN2A and MC1R variants. The series included patients with multiple melanoma (MM, *n* = 78), from melanoma-prone families (FM, *n* = 74), with melanoma and other tumors (MN, *n* = 15), pediatric patients (PM, *n* = 22) and sporadic melanoma cases (SM, *n* = 59). Table 1 reports the genotype results of the studied patients. Genotyping of MC1R gene revealed that most patients carried MC1R variants, and a similar frequency of carriers was observed in MM (72%), FM (72%), MN (73%) and SM (70%), while they were fewer in the PM cohort (55%); in addition, the MM patients showed a high fraction of carriers of two variants compared to the other groups. When the combination of variants was analyzed, assigning each variant to the high risk ‘R’ or the low risk ‘r’ groups according to the established classification, carriers of one ‘r’ variant were the most common in the FM, NM and SM groups, while the MM patients showed a prevalence of ‘R/r’ genotype (Table 1). 

The screening for the MITF-E318K variant identified seven carriers, two in the MM patients’ group, three FM patients, and one in the NM and the SM cohorts (Table 2); three out of seven carried CDKN2A G101W, and 5/7 carried MC1R ‘R’ variants. Of the two MM carriers without positive family history for CM, one carried the CDKN2A G101W pathogenic variant and the ‘R/r’ MC1R genotype (R151C; I155T; T314T), while the second patient was wild type for CDKN2A and carried a synonymous MC1R variant (Q233Q). The three MITF-E318K carriers of the FM group were from two different families, two were the probands and one was an affected family member. Their pedigree is reported in Figure S2: the G101W CDKN2A mutation was detected in one family, while all patients were carriers of high risk MC1R alleles. Notably, these patients developed CM at a young age (mean 31 years, Table 2), as previously reported in several other families with MITF-E318K mutation [21,29]. The MN carrier of MITF-E318K was a melanoma patient developing papillary thyroid carcinoma, a tumor type known to be associated with melanoma [47] but not described before in E318K MITF carriers with melanoma; this patient carried a ‘R’ genotype for MC1R. In contrast, the SM MITF-E318K carrier (EW29/Pt5) showed no association with CDKN2A or MC1R variants (Table 2).

### 3.2. Germline and Somatic Alterations of the MITF-E318K SM Carrier EW29/Pt5

The patient showed a clinical disease course with lymph node presentation and unknown primary site, a rapid progression to disseminated stage IVM1c disease and to death despite treatment with BRAF/MEK inhibitors and subsequent immunotherapy with ipilimumab and then with nivolumab. Exome sequencing data were available for blood and matched tumor DNA from four metastatic lesions of this patient, two excised before (Mel5-pre1 and Mel5-pre2) and two after therapy (Mel5-post1 and Mel5-post2) with BRAF/MEK inhibitors. To gain further insights into the germline/somatic genetic pattern linked to MITF-E318K in a SM carrier, we screened a list of known melanoma susceptibility genes and other putative cancer predisposition genes (Table S1) to investigate whether this patient carried additional variants associated with melanoma and whether new somatic variants or somatic second hits (mutations or CNAs) were present in tumors. After the application of variant filtering rules (described in Section 2), no variants in other high penetrance melanoma predisposition genes and no high penetrance unambiguous loss of function mutations in other major cancer susceptibility genes were detected in this individual. The analysis identified only variants of unknown significance in ATM and FANCI genes (Table S2).

**Figure 1:**
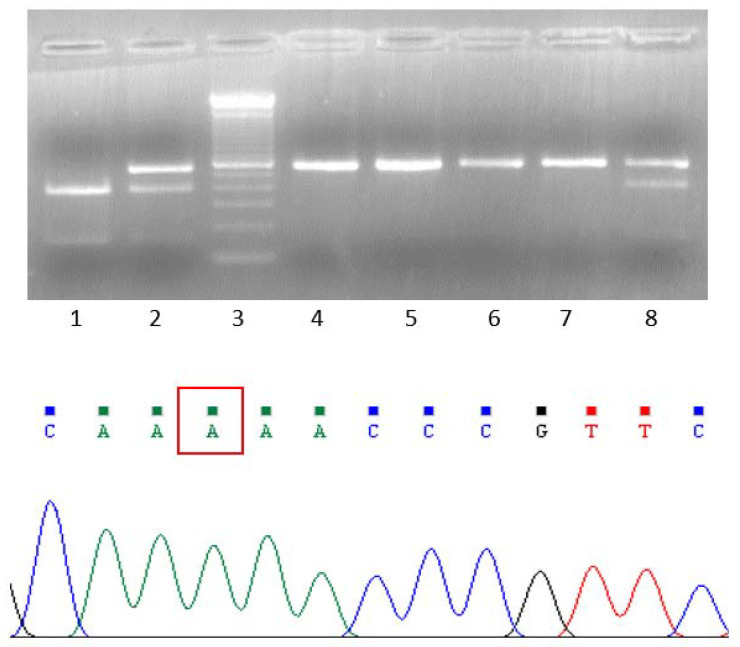
Melanoma tumors showing homozygosity of MITF-E318K variant in patient EW29/Pt5. Electrophoresis of the PCR-RFLP for the detection of MITF-E318K variant in melanoma lesions of EW29/Pt5. Lane 1: negative wild type control; lane 2: positive control heterozygous for MITF-E318K; lane 3: molecular weight marker 110 bp; lanes 4–7: DNA from the four patient’s tumor samples showing homozygosity for the variant. The homozygous mutated sequence is shown in the lower panel; lane 8: patient EW29/Pt5 blood DNA showing heterozygous genotype for the variant. In the sequence the mutated base A is indicated.

Somatic analysis indicated that the number of mutated genes assumed to have a disruptive impact in the protein, in common to all lesions, was 179 (Table S3). Fifteen of them were included in Cosmic Cancer Gene Census (Table S3). The BRAF-V600E mutation of pre-therapy tumors that guided patient clinical treatment with BRAF/MEK inhibitors was confirmed by WES and was maintained in the two post-therapy lesions. Regarding CNA, all events, as well as cytobands and affected genes of the four lesions are listed in Table S4. By considering homozygous deletions and high amplifications as biologically relevant for individual genes, homozygous deletion was observed only at 9p21.3 in one pre-therapy lesion (Mel5-pre2) and involved CDKN2A together with additional genes. The same region was deleted in both post-therapy metastases (Mel5-post1 and Mel5-post2) at heterozygous level (Table S4). Amplification of the long arm of chromosome 8 was peculiar of Mel5-pre2 whereas that of 18q23 and of 7q 31.1-31.2-31.31-31.32 was a shared event among the two post-therapy lesions. These copy gains ranged from 3 to 10fold depending on the region or the lesion. The most notable finding was copy number gain of 3p13, which comprised MITF and several long noncoding RNA (lncRNA) including SAMMSON at levels numbers ranging from 23 (Mel5-pre1) to more than 40 (Mel5-pre2 and Mel5-post2) and up to 88 (Mel5-post1) (Table S4). Genotyping of MITF gene indicated that the only MITF allele present in the four metastatic lesions was the mutant one in contrast to the two alleles (wild-type and mutant) displayed in blood DNA (Figure 1). Overall, in this MITF-E318K carrier, the two most relevant somatic events involving additional predisposition genes were at CNA level and included amplification of 3p13 region containing MITF-E318K, with concomitant loss of the wild-type allele in all lesions, and homozygous deletion of a 9p21.3 region containing CDKN2A and other genes in one of the tumor lesions.

### 3.3. Studies on MITF-E318K Carriers from the TCGA SKCM Collection

Six MITF-E318K carriers from the TCGA SKCM collection were recently reported with their CDKN2A status [48]. Similarly to patient EW29/Pt5, all patients had a CDKN2A germline wild-type allele, avoiding possible confounding effects by this gene (Table 3). These patients were genotyped for MC1R [49] and shown to carry V92M, R151C, R160W or D294H variants, with ‘R/r’, ‘R/0′, and ‘r/0′ genotypes (Table 3). Mutations and CNAs affecting the matched tumors of the six SKCM MITF-E318K sporadic melanoma carriers were downloaded from TCGA Pan Cancer Atlas dataset at the cBioPortal for Cancer Genomics repository. In common with EW29/Pt5 metastatic lesions, all shared the BRAF-V600E mutation, whereas DNAH5 was mutated in four and FAT4 in three out of six cases. Frequency of occurrence of mutation for these genes in all SKCM samples of TCGA (*n* = 438) is 53% for BRAF, 57% for DNAH5 and 39% for FAT4. Although the number of MITF-E318K carriers is low, the presence of BRAF mutation in sporadic melanomas of all of them (100% including the multiple lesions of patient EW29/Pt5) is intriguing and deserves confirmation in larger cohorts.

CNAs were available for five out of six samples. Heterozygous deletion of 3p13 region containing MITF was observed in one TCGA sample. Common CNAs were found on chromosome 9p and involved deep deletion of the 9p21.3 region, as shown in Table S5. In particular, 11 genes from this locus (CDKN2A and CDKN2B; MTAP, encoding an enzyme that plays a major role in polyamine metabolism and is important for the salvage of both adenine and methionine; four members of the interferon gene cluster IFNA1, IFNA2, IFNA8, IFNE; the Double-sex and Mab-3 Related Transcription factor DMRTA1; the long non-coding RNAs LINC01239, MIR31HG and CDKN2B-AS1) displayed homozygous deletion in two out of five TCGA samples. Heterozygous deletion of these genes was observed in all other samples (Table S5). In one sample with heterozygous deletion of the 9p21.3 region, the tumor suppressor MTAP was found deleted at homozygous level (Table S5). Thus, homozygous deletions of MTAP affected four of nine MITF-E318K patients (44%), a percentage higher than that observed in non-MITF-E318K SKCM carriers with only 19% of affected samples.

## 4. Discussion

Genotyping for MITF has been proposed in addition to CDKN2A/CDK4 testing for familial and multiple CM patients, and MITF-E318K carriers are encouraged to follow melanoma prevention programs and dermatologic surveillance. The screening for MITF-E318K variant in our clinical cohort identified seven carriers with an overall frequency that is in line with the rare occurrence of the variant in the Italian population [28,29] as well as in other European countries close to Italy [17,50,51]. The potential interaction between inherited genetic variation in the MC1R gene, a primary regulator of skin pigmentation associated with increased risk of melanoma, and MITF-E318K has been reported but not confirmed in all studies [16,18,52,53]. The distribution of the MC1R variants indeed did not differ significantly in a large case set where 22 MITF carriers were compared to 962 non-carries [24]. MC1R ‘R’ and ‘r’ variants, associated with red hair and fair skin sensitive to ultra-violet radiation and/or to melanoma development, were detected here at frequencies similar to previous reports [54,55]. According to previous observations in the Italian population, a considerable fraction of MM (28%) and of FM (41%) showed CDKN2A pathogenic mutations, while they were very rare or absent in the other tested cohorts (Table 1) [27,31,56]. The most common mutation was G101W, although other known variants were also detected. In accordance with these findings, CDKN2A-G101W missense mutation was found only in the FM and MM groups of MITF-E318K carriers of our cohort.

No CDKN2A or MC1R variants were observed for the single MITF-E318K SM carrier (EW29/Pt5). WES analysis indicated that this patient displayed no variants in other high penetrance melanoma predisposition genes and no high penetrance unambiguous loss of function mutations in other major cancer susceptibility genes but displayed germline variants ATM G2023R and FANCI M525V of unknown significance (Table S2). The association between variants in ATM and melanoma risk is not well established although ATM is associated with a low-to-intermediate risk of other cancers [57]. The occurrence of ATM variants other than G2023R in family probands negative for CDKN2A and CDK4, one being a MITF-E318K carrier, was previously reported [29]. Furthermore, in melanoma-prone families with pancreatic cancers, ATM variants were only observed in patients without germline CDKN2A mutations [58]. FANCI is a Fanconi anemia (FA) protein and mutations in FA genes predispose to tumor development, as shown not only for hereditary breast and ovarian cancer, but also for early-onset/familial prostate carcinoma and acute myeloid leukemia [59]. The FANCI missense variant M525V (rs144908351) is recorded as uncertain significance and was previously reported in ovarian and breast cancer patients [60,61].

Scant information is available on the effect of germline MITF-E318K mutation in melanoma progression and on the genomic landscape of tumors which develop in MITF-E318K carriers. The application of WES to multiple metastatic lesions of the SM carrier EW29/Pt5 revealed somatic deletion of the wild-type MITF allele and amplification of the MITF-E318K one. This variant allele displayed the highest level of amplification with a range varying, in the different lesions, from 23 to 88 copies. Somatic MITF-E318K homozygosity was previously reported in one CM case developing multiple melanomas [20] and in two pheochromocytoma patients showing highly aggressive disease [62]. Whether amplification of the MITF-E318K mutant associates with aggressive disease progression and with resistance to BRAF/MEK inhibitors remains to be confirmed in other studies.

MITF-E318K sporadic CM carriers within the SKCM TCGA cohort were all CDKNA wild type at the germline level, as was EW29/Pt5. The analysis of *in silico* data of all MITF-E318K tumor samples revealed a common genetic pattern for further characterizations and potential therapeutic opportunities. In fact, all tumors displayed the BRAF-V600E mutation. Homozygous loss of MTAP at 9p21.3 locus was observed in four out of nine samples, and, in three of them, that included one of the lesions of EW29/Pt5, the MTAP gene was co-deleted with CDKN2A and other nine adjacent genes. Loss of MTAP determines the intracellular accumulation of its substrate methylthioadenosine, which specifically and potently inhibits the enzymatic activity of protein arginine methyltransferase 5 (PRMT5). In MTAP deleted cancers, PRMT5 and methionine adenosyltransferase II α (MAT2A) enzymes were identified as synthetic lethal targets [63]. Cells from an MTAP-deleted colon carcinoma cell line displayed reduced PRMT5 methylation activity and increased sensitivity to PRMT5 depletion. Furthermore, the cells showed reduced proliferation and PRMT5 methylation activity upon depletion of MAT2A. A selective MAT2A inhibitor able to block the growth of MTAP-null cells both in tissue culture and xenograft tumors has been recently proposed for assessment in phase 1 clinical trial [64].

## 5. Conclusions

In conclusion, we characterized MITF-E318K in a large clinical cohort of melanomas and portrayed in detail germline and somatic alterations in predisposition genes in a SM carrier. Data from this patient and from cases included in TCGA suggests that, in addition to BRAF mutations, homozygous deletion of the MTAP gene, accompanied or not by 10 other genes at 9p21.3, is the most common CNA among MITF-E318K carriers. Our results highlight the importance of performing somatic and germline tests in the future for melanoma as well as for other types of cancer. Analysis of cancer-related genes in paired germline and tumor DNA samples can lead to increased detection of clinically significant alterations and improve risk prediction. Additionally, as in the cases described here, these alterations can unveil cancer dependencies that have the potential to inform novel therapeutic strategies.

## Figures and Tables

**Table 1:** Genetic characteristics of the studied cohorts of CM patients.

Melanoma Cohorts ^a^	MM	FM	NM	SM	PM
N cases (tot 248)	78	74	15	59	22
Females–males	39–39	47–27	9–6	20–39	9–13
Age at diagnosis (range; mean; median)	19–86; 47; 44	19–84; 45; 42	31–80; 57; 60	23–90; 51; 51	1–18; 9; 9
Carriers of E318K MITF variant (tot 7; 4.7%)	2 (2.6%)	3 (4.0%)	1 (6.7%)	1 (1.7%)	0
Carriers of CDKN2A variants	15 (29%)	28 (37%)	0	1 (1.7%)	1 (4.6%)
G101W	8	19	0	1	0
Other CDKN2A variants ^b^	7	9	0	0	1
Carriers of MC1R variants ^c^	56 (72%)	53 (72%)	11 (73%)	41 (70%)	12 (55%)
≥2	28 (36%)	19 (26%)	4 (27%)	12 (20%)	3 (14%)
1	28 (36%)	34 (46%)	8 (53%)	29 (49%)	9 (41%)
0 (wt)	22 (28%)	21 (28%)	4 (27%)	18 (31%)	10 (45%)
R/R	4 (5%)	8 (11%)	1 (7%)	2 (3%)	0
R/r	23 (29%)	8 (11%)	2 (13%)	7 (12%)	1 (5%)
R/0	14 (18%)	15 (20%)	3 (20%)	11 (19%)	3 (14%)
r/r	1 (1%)	3 (4%)	1 (7%)	3 (5%)	2 (9%)
r/0	14 (18%)	19 (26%)	5 (33%)	18 (31%)	6 (27%)

^a^ MM, multiple melanoma; FM, familial melanoma; NM, melanoma and other tumors; SM, sporadic melanoma; PM, pediatric melanoma. FM, MM, NM and PM patients were also tested for CDK4 variants, and all resulted negative for CDK4 mutations. ^b^ Other CDKN2A variants detected were in MM, R24P; S56I; Q70H; N71I; E88G; V126D; G150V; IVS1 + 37C/G; in FM, R24P; R46R; P48T; R99P; V126D; H142R; in PM, CDKN2A locus deletion [30]. ^c^ MC1R variants D84E; R142H; R151C; Y152H; R160W; D294H were classified as ‘R’; MC1R variants V60L; V92M; I155T; R163Q were classified as ‘r’ [31,32]. Other rare MC1R variants of unknown significance were defined ‘r’ (A64T; T95M; G248V; P256S; N279K; D294K). Synonymous variants (T314T; Q233Q; S293S; C151C; L263L) were included in 0 (wt).

**Table 2:** Characteristics of the seven CM carriers of MITF-E318K variant.

Pt ID	Sex/Age at Diagnosis	Pts Group	No. of Melanoma	Other Tumors	CDKN2A Status	MC1R Variants	MC1R Genotype
2844	F/22	FM	4	No	G101W	D294H	R/0
2845	F/30	FM *	1	No	G101W	R151C; V92M; (T314T)	R/r
2907	M/23	FM	2	No	wt	R151C; V60L	R/r
GM89	F/43	MM	2	No	wt	(Q233Q)	0 (cons)
GM92	M/34	MM	3	No	G101W	R151C; I155T; (T314T)	R/r
3008	M/22	MN	2	ca. thyroid	wt	R160W	R/0
EW29/Pt5	M/47	SM	-	No	wt	wt	0

FM, family index case; FM *, affected family member; MM, multiple melanoma; MN, melanoma and other tumors (thyroid carcinoma); SM, sporadic melanoma. The pedigree diagrams of the FM are reported in Figure S2. The mean age at diagnosis is 31 years.

**Table 3:** MITF-E318K carriers of SKCM-TCGA Pan Cancer Atlas cohort.

Patients			Genes			Genotype	Melanoma Features		
Patient-ID	Sex	Age ^a^	MITF ^b^	CDKN2A ^b^	MC1R ^c^	MC1R ^c^	Sample Type	Tumor Disease Anatomic Site	BRAF Status
TCGA-D3-A1Q5	M	60	E318K	wt	V92M, R160W	R/r	Metastasis	Regional lymph node	V600E
TCGA-D3-A1Q6	M	55	E318K	wt	D294H	R/0	Metastasis	Subcutaneous	V600E
TCGA-EE-A2M8	F	54	E318K	wt	R151C	r/0	Metastasis	Regional lymph node	V600E
TCGA-FS-A1ZS	M	54	E318K	wt	V92M	r/0	Metastasis	Regional lymph node	V600E
TCGA-W3-AA21	M	26	E318K	wt	NA	NA	Metastasis	Regional lymph node	V600E
TCGA-GF-A2C7	M	48	E318K	wt	wt	0	Primary	Head and neck	V600E

^a^ Age at diagnosis, the mean is 49.5 years. ^b^ Status of MITF and CDKN2A as previously reported [48]. ^c^ MC1R genotype as previously reported [49].

## Supplementary Materials

The following are available online at https://www.mdpi.com/article/10.3390/genes12091440/s1, Figure S1: Description of the RLFP method for the detection of the MITF-E318K variant, Figure S2: Pedigrees of the two CM families carrying the MITF-E318K variant, Table S1: Cancer predisposition genes selected for germline analysis, Table S2: Germline missense variants in SM patient EW29/Pt5, Table S3: Somatic variants shared by the different metastatic lesions of SM patient EW29/Pt5, Table S4: Amplified or deleted chromosomal regions and genes in the different metastatic lesions of SM patient EW29/Pt5, Table S5: Homozygous deletions for genes at 9p21.3 chromosomal region in MITF-E318K carriers, Table S6: Copy number alterations for genes at 9p21.3 and 3p13 chromosomal regions in all MITF-E318K carriers.

## References

[1] Goding C.R., Arnheiter H. (2019). MITF—The first 25 years. Genes Dev..

[2] Li X., Mao W., Chen J., Goding C.R., Cui R., Xu Z.-X., Miao X. (2021). The protective role of MC1R in chromosome stability and centromeric integrity in melanocytes. Cell Death Discov..

[3] Hoek K.S., Goding C.R. (2010). Cancer stem cells versus phenotype-switching in melanoma. Pigment. Cell Melanoma Res..

[4] Sensi M., Catani M., Castellano G., Nicolini G., Alciato F., Tragni G., De Santis G., Bersani I., Avanzi G., Tomassetti A. (2011). Human Cutaneous Melanomas Lacking MITF and Melanocyte Differentiation Antigens Express a Functional Axl Receptor Kinase. J. Investig. Dermatol..

[5] Giuliano S., Cheli Y., Ohanna M., Bonet C., Beuret L., Bille K., Loubat A., Hofman V., Hofman P., Ponzio G. (2010). Microphthalmia-Associated Transcription Factor Controls the DNA Damage Response and a Lineage-Specific Senescence Program in Melanomas. Cancer Res..

[6] Garraway L.A., Widlund H., Rubin M., Getz G., Berger A.J., Ramaswamy S., Beroukhim R., Milner J.D.A., Granter S.R., Du J. (2005). Integrative genomic analyses identify MITF as a lineage survival oncogene amplified in malignant melanoma. Nat. Cell Biol..

[7] Müller J., Krijgsman O., Tsoi J., Robert L., Hugo W., Song C., Kong X., Possik P.A., Cornelissen-Steijger P.D.M., Foppen M.H.G. (2014). Low MITF/AXL ratio predicts early resistance to multiple targeted drugs in melanoma. Nat. Commun..

[8] Smith M.P., Ferguson J., Arozarena I., Hayward R., Marais R., Chapman A., Hurlstone A., Wellbrock C. (2012). Effect of SMURF2 Targeting on Susceptibility to MEK Inhibitors in Melanoma. J. Natl. Cancer Inst..

[9] Sánchez-Del-Campo L., Martí-Díaz R., Montenegro M.F., González-Guerrero R., Hernández-Caselles T., Martínez-Barba E., Piñero-Madrona A., Cabezas-Herrera J., Goding C.R., Rodríguez-López J.N. (2021). MITF induces escape from innate immunity in melanoma. J. Exp. Clin. Cancer Res..

[10] Roesch A., Paschen A., Landsberg J., Helfrich I., Becker J.C., Schadendorf D. (2016). Phenotypic tumour cell plasticity as a resistance mechanism and therapeutic target in melanoma. Eur. J. Cancer.

[11] Bertolotto C., Lesueur F., Giuliano S., Strub T., De Lichy M., Bille K., Dessen P., D’Hayer B., Mohamdi H., Remenieras A. (2011). A SUMOylation-defective MITF germline mutation predisposes to melanoma and renal carcinoma. Nature.

[12] Yokoyama S., Woods S.L., Boyle G.M., Aoude L.G., MacGregor S., Zismann V., Gartside M., Cust A.E., Haq R., Harland M. (2011). A novel recurrent mutation in MITF predisposes to familial and sporadic melanoma. Nature.

[13] Zhao X. (2018). SUMO-Mediated Regulation of Nuclear Functions and Signaling Processes. Mol. Cell.

[14] Roider E.M., Fisher D.E. (2014). The impact of MITF on melanoma development: News from bench and bedside. J. Investig. Dermatol..

[15] Paillerets B.B.-D., Lesueur F., Bertolotto C. (2014). A germline oncogenic MITF mutation and tumor susceptibility. Eur. J. Cell Biol..

[16] Berwick M., MacArthur J., Orlow I., Kanetsky P., Begg C.B., Luo L., Reiner A., Sharma A., Armstrong B.K., Kricker A. (2014). MITF E318K’s effect on melanoma risk independent of, but modified by, other risk factors. Pigment. Cell Melanoma Res..

[17] Potrony M., Puig-Butille J.A., Aguilera P., Badenas C., Tell-Marti G., Carrera C., Del Pozo L.J., Conejo-Mir J., Malvehy J., Puig S. (2016). Prevalence ofMITFp.E318K in Patients with Melanoma Independent of the Presence ofCDKN2ACausative Mutations. JAMA Dermatol..

[18] Sturm R.A., Fox C., McClenahan P., Jagirdar K., Ibarrola-Villava M., Banan P., Abbott N.C., Ribas G., Gabrielli B., Duffy D.L. (2014). Phenotypic Characterization of Nevus and Tumor Patterns in MITF E318K Mutation Carrier Melanoma Patients. J. Investig. Dermatol..

[19] Bonet C., Luciani F., Ottavi J.-F., Leclerc J., Jouenne F.-M., Boncompagni M., Bille K., Hofman V., Bossis G., De Donatis G.M. (2017). Deciphering the Role of Oncogenic MITFE318K in Senescence Delay and Melanoma Progression. J. Natl. Cancer Inst..

[20] Bassoli S., Pellegrini C., Longo C., Di Nardo L., Farnetani F., Cesinaro A.M., Pellacani G., Fargnoli M.C. (2018). Clinical, dermoscopic, and confocal features of nevi and melanomas in a multiple primary melanoma patient with the MITF p.E318K homozygous mutation. Melanoma Res..

[21] Ghiorzo P., Pastorino L., Queirolo P., Bruno W., Tibiletti M.G., Nasti S., Andreotti V., Paillerets B.B.-D., Scarrà G.B., Genoa Pancreatic Cancer Study Group (2012). Prevalence of the E318K MITF germline mutation in Italian melanoma patients: Associations with histological subtypes and family cancer history. Pigment. Cell Melanoma Res..

[22] Guhan S.M., Artomov M., McCormick S., Njauw C.-N., Stratigos A.J., Shannon K., Ellisen L.W., Tsao H. (2020). Cancer risks associated with the germline MITF(E318K) variant. Sci. Rep..

[23] Pollio A., Tomasi A., Seidenari S., Pellacani G., Rodolfo M., Frigerio S., Maurichi A., Turchetti D., Bassoli S., Ruini C. (2013). Malignant and benign tumors associated with multiple primary melanomas: Just the starting block for the involvement ofMITF, PTENandCDKN2Ain multiple cancerogenesis?. Pigment. Cell Melanoma Res..

[24] Ciccarese G., Dalmasso B., Bruno W., Queirolo P., Pastorino L., Andreotti V., Spagnolo F., Tanda E., Ponti G., Italian Melanoma Intergroup (I.M.I.) (2020). Clinical, pathological and dermoscopic phenotype of MITF p.E318K carrier cutaneous melanoma patients. J. Transl. Med..

[25] Dika E., Patrizi A., Rossi C., Turchetti D., Miccoli S., Ferracin M., Veronesi G., Scarfì F., Lambertini M. (2020). Clinical histopathological features and CDKN2A/CDK4/MITF mutational status of patients with multiple primary melanomas from Bologna: Italy is a fascinating but complex mosaic. G. Ital. Dermatol. Venereol..

[26] Lambertini M., Mussi M., Dika E. (2021). Nodular melanoma in an MITF p.E318K carrier patient: The Wolf in Little Red Riding Hood. Australas. J. Dermatol..

[27] Bruno W., Pastorino L., Ghiorzo P., Andreotti V., Martinuzzi C., Menin C., Elefanti L., Stagni C., Vecchiato A., Rodolfo M. (2016). Multiple primary melanomas (MPMs) and criteria for genetic assessment: MultiMEL, a multicenter study of the Italian Melanoma Intergroup. J. Am. Acad. Dermatol..

[28] De Simone P., Bottillo I., Valiante M., Iorio A., De Bernardo C., Majore S., D’Angelantonio D., Valentini T., Sperduti I., Piemonte P. (2020). A Single Center Retrospective Review of Patients from Central Italy Tested for Melanoma Predisposition Genes. Int. J. Mol. Sci..

[29] Pastorino L., Andreotti V., Dalmasso B., Vanni I., Ciccarese G., Mandalà M., Spadola G., Pizzichetta M.A., Ponti G., Tibiletti M.G. (2020). Insights into Genetic Susceptibility to Melanoma by Gene Panel Testing: Potential Pathogenic Variants in ACD, ATM, BAP1, and POT1. Cancers.

[30] Frigerio S., Disciglio V., Manoukian S., Peissel B., Della Torre G., Maurichi A., Collini P., Pasini B., Gotti G., Ferrari A. (2014). A large de novo9p21.3 deletion in a girl affected by astrocytoma and multiple melanoma. BMC Med. Genet..

[31] Kanetsky P.A., Rebbeck T.R., Hummer A.J., Panossian S., Armstrong B.K., Kricker A., Marrett L.D., Millikan R.C., Gruber S.B., Culver H.A. (2006). Population-Based Study of Natural Variation in the Melanocortin-1 Receptor Gene and Melanoma. Cancer Res..

[32] John P.R., Ramsay M. (2002). Four novel variants in MC1R in red-haired South African individuals of European descent: S83P, Y152X, A171D, P256S. Hum. Mutat..

[33] Daniotti M., Ferrari A., Frigerio S., Casieri P., Miselli F., Zucca E., Collini P., Della Torre G., Manoukian S., Peissel B. (2009). Cutaneous Melanoma in Childhood and Adolescence Shows Frequent Loss of INK4A and Gain of KIT. J. Investig. Dermatol..

[34] Bertoldi L., Forcato C., Vitulo N., Birolo G., De Pascale F., Feltrin E., Schiavon R., Anglani F., Negrisolo S., Zanetti A. (2017). QueryOR: A comprehensive web platform for genetic variant analysis and prioritization. BMC Bioinform..

[35] Read J., Wadt K.A.W., Hayward N.K. (2015). Melanoma genetics. J. Med. Genet..

[36] Huang K.-L., Mashl R.J., Wu Y., Ritter D.I., Wang J., Oh C., Paczkowska M., Reynolds S., Wyczalkowski M.A., Oak N. (2018). Pathogenic Germline Variants in 10,389 Adult Cancers. Cell.

[37] Landi M.T., Bishop D.T., MacGregor S., Machiela M.J., Stratigos A.J., Ghiorzo P., Brossard M., Calista D., Choi J., Fargnoli M.C. (2020). Genome-wide association meta-analyses combining multiple risk phenotypes provide insights into the genetic architecture of cutaneous melanoma susceptibility. Nat. Genet..

[38] De Summa S., Guida M., Tommasi S., Strippoli S., Pellegrini C., Fargnoli M.C., Pilato B., Natalicchio I., Guida G., Pinto R. (2016). Genetic profiling of a rare condition: Co-occurrence of albinism and multiple primary melanoma in a caucasian family. Oncotarget.

[39] Christodoulou E., Van Doorn R., Visser M., Teunisse A., Versluis M., Van Der Velden P., Hayward N., Jochemsen A., Gruis N. (2019). NEK11 as a candidate high-penetrance melanoma susceptibility gene. J. Med. Genet..

[40] Campos C., Fragoso S., Luís R., Pinto F., Brito C., Esteves S., Pataco M., Santos S., Machado P., Vicente J.B. (2020). High-Throughput Sequencing Identifies 3 Novel Susceptibility Genes for Hereditary Melanoma. Genes.

[41] Sondka Z., Bamford S., Cole C.G., Ward S.A., Dunham I., Forbes S.A. (2018). The COSMIC Cancer Gene Census: Describing genetic dysfunction across all human cancers. Nat. Rev. Cancer.

[42] Landrum M.J., Lee J.M., Riley G.R., Jang W., Rubinstein W.S., Church D.M., Maglott D.R. (2014). ClinVar: Public archive of relationships among sequence variation and human phenotype. Nucleic Acids Res..

[43] Mayakonda A., Lin D.-C., Assenov Y., Plass C., Koeffler H.P. (2018). Maftools: Efficient and comprehensive analysis of somatic variants in cancer. Genome Res..

[44] D’Aurizio R., Pippucci T., Tattini L., Giusti B., Pellegrini M., Magi A. (2016). Enhanced copy number variants detection from whole-exome sequencing data using EXCAVATOR2. Nucleic Acids Res..

[45] Cerami E., Gao J., Dogrusoz U., Gross B.E., Sumer S.O., Aksoy B.A., Jacobsen A., Byrne C.J., Heuer M.L., Larsson E. (2012). The cBio Cancer Genomics Portal: An Open Platform for Exploring Multidimensional Cancer Genomics Data. Cancer Discov..

[46] Gao J., Aksoy B.A., Dogrusoz U., Dresdner G., Gross B., Sumer S.O., Sun Y., Jacobsen A., Sinha R., Larsson E. (2013). Integrative Analysis of Complex Cancer Genomics and Clinical Profiles Using the cBioPortal. Sci. Signal..

[47] Goggins W., Daniels G.H., Tsao H. (2006). Elevation of thyroid cancer risk among cutaneous melanoma survivors. Int. J. Cancer.

[48] Aoude L.G., Bonazzi V.F., Brosda S., Patel K., Koufariotis L.T., Oey H., Nones K., Wood S., Pearson J.V., Lonie J.M. (2020). Pathogenic germline variants are associated with poor survival in stage III/IV melanoma patients. Sci. Rep..

[49] Espinoza C.D.R., Roberts N., Chen S., Leacy F.P., Alexandrov L.B., Pornputtapong N., Halaban R., Krauthammer M., Cui R., Bishop D.T. (2016). Germline MC1R status influences somatic mutation burden in melanoma. Nat. Commun..

[50] Muller C., Wendt J., Rauscher S., Burgstaller-Muehlbacher S., Sunder-Plassmann R., Scheurecker C., Richtig E., Fae I., Fischer G., Pehamberger H. (2016). Characterization of patients at high risk of melanoma in Austria. Br. J. Dermatol..

[51] Mangas C., Potrony M., Mainetti C., Bianchi E., Merlani P.C., Eberhardt A.M., Maspoli-Postizzi E., Marazza G., Marcollo-Pini A., Pelloni F. (2016). Genetic susceptibility to cutaneous melanoma in southern Switzerland: Role of CDKN2A, MC1R and MITF. Br. J. Dermatol..

[52] Landi M.T., Kanetsky P.A., Tsang S., Gold B., Munroe D., Rebbeck T., Swoyer J., Ter-Minassian M., Hedayati M., Grossman L. (2005). MC1R, ASIP, and DNA Repair in Sporadic and Familial Melanoma in a Mediterranean Population. J. Natl. Cancer Inst..

[53] Palmer J.S., Duffy D., Box N., Aitken J., O’Gorman L.E., Green A.C., Hayward N.K., Martin N., Sturm R.A. (2000). Melanocortin-1 Receptor Polymorphisms and Risk of Melanoma: Is the Association Explained Solely by Pigmentation Phenotype?. Am. J. Hum. Genet..

[54] Pasquali E., Garcia-Borron J.C., Fargnoli M.C., Gandini S., Maisonneuve P., Bagnardi V., Specchia C., Cornelius L.A., Kayser M., Nijsten T. (2014). MC1Rvariants increased the risk of sporadic cutaneous melanoma in darker-pigmented Caucasians: A pooled-analysis from the M-SKIP project. Int. J. Cancer.

[55] Wendt J., Rauscher S., Burgstaller-Muehlbacher S., Fae I., Fischer G., Pehamberger H., Okamoto I. (2016). Human Determinants and the Role of Melanocortin-1 Receptor Variants in Melanoma Risk Independent of UV Radiation Exposure. JAMA Dermatol..

[56] Pellegrini C., Raimondi S., Di Nardo L., Ghiorzo P., Menin C., Manganoni M.A., Palmieri G., Guida G., Quaglino P., Stanganelli I. (2021). Melanoma in Children and Adolescents: Analysis of Susceptibility Genes in 123 Italian Patients. JEADV.

[57] Van Os N., Roeleveld N., Weemaes C., Jongmans M., Janssens G.O.R.J., Taylor A., Hoogerbrugge N., Willemsen M. (2016). Health risks for ataxia-telangiectasia mutated heterozygotes: A systematic review, meta-analysis and evidence-based guideline. Clin. Genet..

[58] Yang X.R., Rotunno M., Xiao Y., Ingvar C., Helgadottir H., Pastorino L., Van Doorn R., Bennett H., Graham C., Sampson J.N. (2016). Multiple rare variants in high-risk pancreatic cancer-related genes may increase risk for pancreatic cancer in a subset of patients with and without germline CDKN2A mutations. Qual. Life Res..

[59] Del Valle J., Rofes P., Moreno-Cabrera J.M., López-Dóriga A., Belhadj S., Vargas-Parra G., Teulé À., Cuesta R., Muñoz X., Campos O. (2020). Exploring the Role of Mutations in Fanconi Anemia Genes in Hereditary Cancer Patients. Cancers.

[60] Wilhite T.J., Youland R.S., Tian S., Finley R.R., Sarkaria J.N., Corbin K.S. (2019). Pathogenic Germ Line Variants in a Patient with Severe Toxicity from Breast Radiotherapy. Clin. Breast Cancer.

[61] Fierheller C.T., Guitton-Sert L., Alenezi W.M., Revil T., Oros K.K., Bedard K., Arcand S.L., Serruya C., Behl S., Meunier L. (2020). The genetic analysis of a founder Northern American population of European descent identifies FANCI as a candidate familial ovarian cancer risk gene. medRxiv.

[62] Castro-Vega L.J., Kiando S.R., Burnichon N., Buffet A., Amar L., Simian C., Berdelou A., Galan P., Schlumberger M., Bouatia-Naji N. (2016). The MITF, p.E318K Variant, as a Risk Factor for Pheochromocytoma and Paraganglioma. J. Clin. Endocrinol. Metab..

[63] Marjon K., Cameron M.J., Quang P., Clasquin M.F., Mandley E., Kunii K., McVay M., Choe S., Kernytsky A., Gross S. (2016). MTAP Deletions in Cancer Create Vulnerability to Targeting of the MAT2A/PRMT5/RIOK1 Axis. Cell Rep..

[64] Konteatis Z., Travins J., Gross S., Marjon K., Barnett A., Mandley E., Nicolay B., Nagaraja R., Chen Y., Sun Y. (2021). Discovery of AG-270, a First-in-Class Oral MAT2A Inhibitor for the Treatment of Tumors with Homozygous MTAP Deletion. J. Med. Chem..

