# Supplementary material for: Genetic Variants and Somatic Alterations Associated with MITF-E318K Germline Mutation in Melanoma Patients
Source: Genes (Basel). 2021 Sep 18;12(9):1440. doi: 10.3390/genes12091440 (PMC8469310; doi:10.3390/genes12091440)
Supplement: Supplementary file 1 [file genes-12-01440-s001.zip › VERGANI SUPPLEMENTARY FIGURES.pdf]

A

5' G A A N N ↓N N T T C 3'
3' C T T N N ↑N N A A G 5'

B

GCTTAAAAGTCCTCTGTGCTCTGCCTATTTCAGTGTTTTATC
TTTACTCTTATTATAGGAACTTGAAATGCAGGCTCGAGCTCA
TGGACTTTCCCTTATTCCATCCACGGGTCTCTGCTCTCCAGAT
TTGGTGAATCGGATCATCAAGCAAGAACCCGTTCTTGAGAA
CTGCAGCCAAGACCTCCTTCAGCATCATGCAGACCTAACCTG
TACAACAACTCTCGATCTCACGGATGGCACCATCACCTTCAA
CAACAACCTCGGAACTGGGACTGAGGCCAACCAAGCCTATA
GTGTCCCCACAAAAATGGGATCCAAACTGGAAGACATCCTG
ATGGACGACACCCTTTCTCCCGTCGGTGTCACTGATCCACTC
CTTTCCTCAGTGTCCCCCGGAGCTTCCAAAACAAGCAGCCG
GAGGAGCAGTATGAGCATGGAAGAGACGGAGCACACTTGT
TAGCGAATCCTCCCTGCACTGCATTCGCACAAACTGCTTCCT
TTCTTGATTCGTAGATTTAATAA**CTTACCTGAAGGGGTTTTC**
TTG

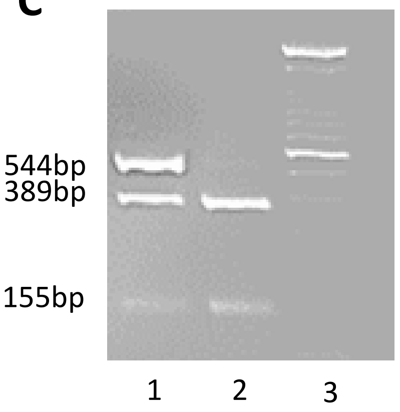

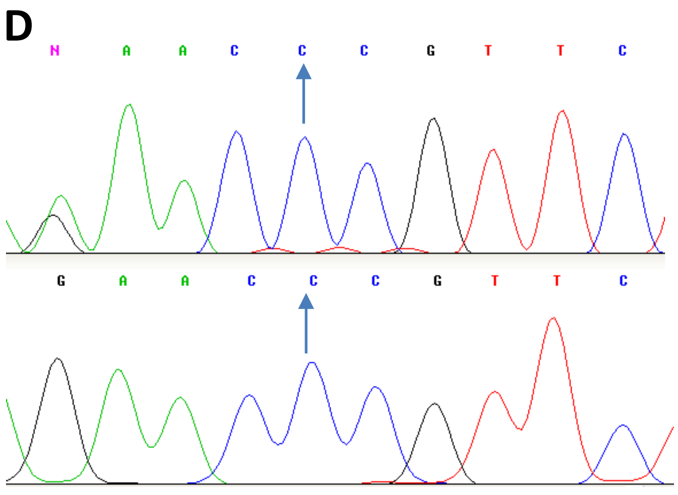

Figure S1. Description of the RLFP method for the detection of the MITF-E318K variant.
(A) Sequence stretch recognized and cut by XmnI restriction enzyme. (B) Sequence of the PCR product: in black bold primers sites, in green nucleotide that changes after the G>A mutation, in red the site recognized by XmnI and in blue its cut site. (C) PCR-RFLP for MITF separated on a 3% agarose gel. The PCR product was cleaved in 20µl final volume reaction mix consisting of 1U of XmnI enzyme, 2 µl Buffer Neb4 10X, 2 µl BSA 10x and 15 µl PCR reaction mixture. The reaction was incubated 2 hours at 37°C before loading. The 544 bp MITF wild-type product after XmnI digestion is cut in two smaller fragments of 389 and 155 bp. Lane 1 shows the heterozygous genotype, lane 2 the wild type genotype, lane 3 the molecular weight marker 110bp. (D) MITF gene mutation sequence G>A indicated as N; the nucleotide indicated by arrows is XmnI cut site.

Family 1

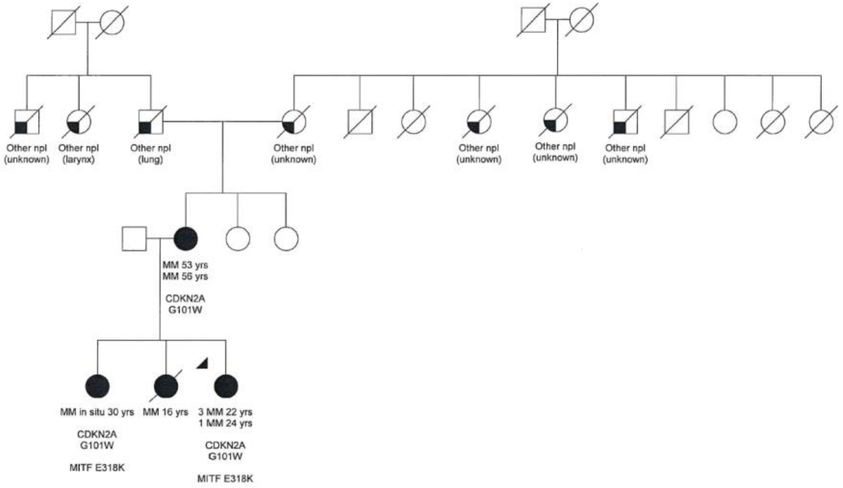

Family 2

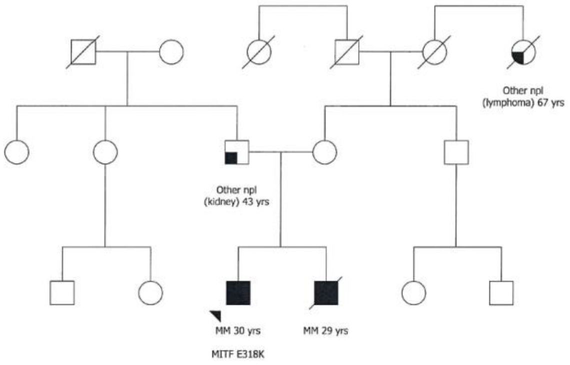

Figure S2. Pedigrees of the two CM families carrying the MITF-E318K variant.

Dark symbol cutaneous melanoma. Cancer type and age at diagnosis are indicated for each symbol and the proband is indicated by the arrow
